# ADVANCING AN INTERDISCIPLINARY SCIENCE OF AGING THROUGH A PRACTICE-BASED DATA SCIENCE APPROACH

**DOI:** 10.1093/geroni/igz038.1786

**Published:** 2019-11-08

**Authors:** Robert Lucero, Ragnhildur Bjarnadottir

**Affiliations:** University of Florida, Gainesville, Florida, United States

## Abstract

Two hundred and fifty thousand older adults die annually in United States hospitals because of iatrogenic conditions (ICs). Clinicians, aging experts, patient advocates and federal policy makers agree that there is a need to enhance the safety of hospitalized older adults through improved identification and prevention of ICs. To this end, we are building a research program with the goal of enhancing the safety of hospitalized older adults by reducing ICs through an effective learning health system. Leveraging unique electronic data and healthcare system and human resources at the University of Florida, we are applying a state-of-the-art practice-based data science approach to identify risk factors of ICs (e.g., falls) from structured (i.e., nursing, clinical, administrative) and unstructured or text (i.e., registered nurse’s progress notes) data. Our interdisciplinary academic-clinical partnership includes scientific and clinical experts in patient safety, care quality, health outcomes, nursing and health informatics, natural language processing, data science, aging, standardized terminology, clinical decision support, statistics, machine learning, and hospital operations. Results to date have uncovered previously unknown fall risk factors within nursing (i.e., physical therapy initiation), clinical (i.e., number of fall risk increasing drugs, hemoglobin level), and administrative (i.e., Charlson Comorbidity Index, nurse skill mix, and registered nurse staffing ratio) structured data as well as patient cognitive, environmental, workflow, and communication factors in text data. The application of data science methods (i.e., machine learning and text-mining) and findings from this research will be used to develop text-mining pipelines to support sustained data-driven interdisciplinary aging studies to reduce ICs.

